# Supplementary material for: Pyruvate Dehydrogenase Kinase 1 inhibition mediated oxidative phosphorylation enhancement in cartilage promotes osteoarthritis progression
Source: BMC Musculoskelet Disord. 2023 Jul 20;24:597. doi: 10.1186/s12891-023-06585-6 (PMC10357736; doi:10.1186/s12891-023-06585-6)
Supplement: Supplementary file 1 — Additional file 1. [file 12891_2023_6585_MOESM1_ESM.zip › MF.Gsea.1653627316242/GOMF_PROTEIN_CARRIER_CHAPERONE.html]

Details for gene set GOMF\_PROTEIN\_CARRIER\_CHAPERONE[GSEA]

|  || Dataset | 55457\_collapsed\_to\_symbols.cls.cls #OA\_versus\_normal.cls.cls #OA\_versus\_normal\_repos |
| Phenotype | cls.cls#OA\_versus\_normal\_repos |
| Upregulated in class | OA |
| GeneSet | GOMF\_PROTEIN\_CARRIER\_CHAPERONE |
| Enrichment Score (ES) | 0.5311304 |
| Normalized Enrichment Score (NES) | 1.6134322 |
| Nominal p-value | 0.015765766 |
| FDR q-value | 0.13543369 |
| FWER p-Value | 0.979 |
Table: GSEA Results Summary

  

Fig 1: Enrichment plot: GOMF\_PROTEIN\_CARRIER\_CHAPERONE      
 Profile of the Running ES Score & Positions of GeneSet Members on the Rank Ordered List

  

| SYMBOL | TITLE | RANK IN GENE LIST | RANK METRIC SCORE | RUNNING ES | CORE ENRICHMENT || 1 | EMC9 | ER membrane protein complex subunit 9 [Source:HGNC Symbol;Acc:HGNC:20273] | 276 | 0.768 | 0.1180 | Yes |
| 2 | EMC7 | ER membrane protein complex subunit 7 [Source:HGNC Symbol;Acc:HGNC:24301] | 468 | 0.673 | 0.2253 | Yes |
| 3 | EMC1 | ER membrane protein complex subunit 1 [Source:HGNC Symbol;Acc:HGNC:28957] | 1067 | 0.502 | 0.2699 | Yes |
| 4 | EMC2 | ER membrane protein complex subunit 2 [Source:HGNC Symbol;Acc:HGNC:28963] | 1096 | 0.497 | 0.3579 | Yes |
| 5 | EMC3 | ER membrane protein complex subunit 3 [Source:HGNC Symbol;Acc:HGNC:23999] | 1624 | 0.410 | 0.3915 | Yes |
| 6 | CLU | clusterin [Source:HGNC Symbol;Acc:HGNC:2095] | 2083 | 0.352 | 0.4198 | Yes |
| 7 | PEX19 | peroxisomal biogenesis factor 19 [Source:HGNC Symbol;Acc:HGNC:9713] | 2223 | 0.336 | 0.4700 | Yes |
| 8 | TIMM10 | translocase of inner mitochondrial membrane 10 [Source:HGNC Symbol;Acc:HGNC:11814] | 2244 | 0.333 | 0.5289 | Yes |
| 9 | EMC6 | ER membrane protein complex subunit 6 [Source:HGNC Symbol;Acc:HGNC:28430] | 2839 | 0.266 | 0.5311 | Yes |
| 10 | TIMM9 | translocase of inner mitochondrial membrane 9 [Source:HGNC Symbol;Acc:HGNC:11819] | 4603 | 0.104 | 0.4133 | No |
| 11 | OXA1L | OXA1L mitochondrial inner membrane protein [Source:HGNC Symbol;Acc:HGNC:8526] | 5974 | -0.003 | 0.3075 | No |
| 12 | EMC8 | ER membrane protein complex subunit 8 [Source:HGNC Symbol;Acc:HGNC:7864] | 6104 | -0.013 | 0.2998 | No |
| 13 | HSPA8 | heat shock protein family A (Hsp70) member 8 [Source:HGNC Symbol;Acc:HGNC:5241] | 9140 | -0.243 | 0.1084 | No |
| 14 | EMC10 | ER membrane protein complex subunit 10 [Source:HGNC Symbol;Acc:HGNC:27609] | 11497 | -0.501 | 0.0164 | No |
| 15 | BAG3 | BAG cochaperone 3 [Source:HGNC Symbol;Acc:HGNC:939] | 11562 | -0.509 | 0.1038 | No |
Table: GSEA details [plain text format]

  

Fig 2: GOMF\_PROTEIN\_CARRIER\_CHAPERONE      
 Blue-Pink O' Gram in the Space of the Analyzed GeneSet

  

Fig 3: GOMF\_PROTEIN\_CARRIER\_CHAPERONE: Random ES distribution      
 Gene set null distribution of ES for **GOMF\_PROTEIN\_CARRIER\_CHAPERONE**
